# Real-World Analysis of Weight Gain and Body Mass Index Increase Among Patients with HIV-1 Using Antiretroviral Regimen Containing Tenofovir Alafenamide, Tenofovir Disoproxil Fumarate, or Neither in the United States

**DOI:** 10.36469/jheor.2022.31825

**Published:** 2022-02-14

**Authors:** Bruno Emond, Carmine Rossi, Rachel Rogers, Patrick Lefebvre, Marie-Hélène Lafeuille, Prina Donga

**Affiliations:** 1 Analysis Group, Inc., Montréal, Québec, Canada; 2 Janssen Scientific Affairs, LLC., Titusville, New Jersey, USA

**Keywords:** HIV, tenofovir alafenamide, tenofovir disoproxil fumarate, weight gain, body mass index, electronic health records, observational study

## Abstract

**Background:** While some studies among patients with HIV-1 suggest that antiretroviral therapy (ART) regimens containing tenofovir alafenamide (TAF) may be associated with greater weight gain than those not containing TAF, no studies have assessed the relationship between TAF doses and weight change. **Objectives:** To evaluate weight-related outcomes among patients with HIV-1 in the United States initiating ART containing different nucleoside reverse transcriptase inhibitors and doses. **Methods:** A retrospective longitudinal study was conducted using Decision Resources Group’s electronic medical records (July 17, 2017-March 1, 2020). Adult patients with HIV-1 initiating ART (index date) containing TAF 25 mg, TAF 10 mg, tenofovir disoproxil fumarate (TDF), or neither TAF nor TDF on or after July 17, 2018, were included. Changes in weight and body mass index (BMI) from pre-index to 3, 6, 9, and 12 months post-index were compared between cohorts using mean differences obtained from ordinary least squares models adjusted for baseline characteristics. Time-to-weight and BMI increase ≥5% were compared using Cox models adjusted for baseline characteristics. **Results:** Among 1652 eligible patients (TAF 25 mg, n=710; TAF 10 mg, n=303; TDF, n=219; non-TAF/TDF, n=420), the majority (83.2%-99.5%) initiated an integrase strand transfer inhibitor, except for the TDF cohort (45.2%). Patients initiating TAF 25 mg had greater weight or BMI increase across all time points compared with patients initiating TAF 10 mg, TDF, or non-TAF/TDF regimens (mean differences in weight or BMI changes between cohorts at 12 months post-index ranged from 0.78 kg [1.72 lb] to 1.34 kg [2.95 lb] and from 0.77 kg/m2 to 1.95 kg/m2, respectively), although findings were not statistically significant for all comparisons. Compared with TAF 25 mg, time-to-weight and BMI increase ≥5% in the other treatment cohorts were longer (hazard ratios ranged from 0.77 to 0.94), although findings were generally not statistically significant. **Conclusions:** Among a population of patients predominantly initiating integrase strand transfer inhibitors, increases in weight and BMI post-ART initiation were common and appeared to be higher and occur more rapidly among patients receiving TAF 25 mg compared with lower TAF doses or other nucleosides. When considering long-term health consequences, weight gain is an important factor to consider when selecting an ART regimen.

## BACKGROUND

Human immunodeficiency virus (HIV-1) is a chronic, lifelong infectious condition that has been historically associated with high mortality. Since the advent of antiretroviral therapy (ART) in the late 1990s, the life expectancy of patients living with HIV-1 (PLWH) has increased dramatically, in some cases even approaching that of non-HIV populations.^1^ Among the various treatment classes and agents available, integrase strand transfer inhibitor (INSTI)–based ART regimens are recommended by the US Department of Health and Human Services (DHHS) guidelines in most clinical situations. However, for patients at risk of poor adherence or for patients who require rapid initiation of ART before genotypic drug resistance testing results are available, the use of boosted darunavir, a protease inhibitor (PI); bictegravir, an INSTI; or dolutegravir (DTG), an INSTI; is specifically recommended.^2^

Despite advances in HIV-1 therapeutics, all patients will require treatment that may span over many decades due to the chronic nature of the disease. As a result, it is important to balance the clinical benefits against any cumulative risks that may be associated with prolonged exposure to ART regimens.^1^ In particular, weight gain is a factor that may warrant careful consideration when selecting the appropriate ART regimen. According to DHHS guidelines, INSTI-based regimens have been associated with greater weight gain than PI or non-nucleoside reverse transcriptase inhibitor (NNRTI)-based ART regimens.^2^ Given the results from several recent studies, DHHS guidelines revised in December 2019 introduced evidence demonstrating that ART regimens containing the nucleoside reverse transcriptase inhibitor (NRTI) tenofovir alafenamide (TAF) are associated with a greater weight gain than ART regimens containing tenofovir disoproxil fumarate (TDF) among treatment-naïve^2–4^ as well as stable (ie, virologically suppressed)^5^ and nonstable^6–8^ patients switching from a previous ART, especially when combined with an INSTI.^9^ Moreover, current DHHS guidelines acknowledge that TAF has been associated with greater weight gain than abacavir/lamivudine in treatment-naïve PLWH.^2,3^

TAF is available in doses of 25 mg (eg, as part of the single-tablet regimen [STR] bictegravir/emtricitabine/TAF [BIC/FTC/TAF])^10^ and 10 mg (eg, as part of the STR darunavir/cobicistat/FTC/TAF [DRV/c/FTC/TAF])^11^ or elvitegravir/ cobicistat/FTC/TAF (EVG/c/FTC/TAF).^10^ However, no studies to date have assessed the relationship between different doses of TAF (ie, TAF 25 mg, which is used without a booster as part of regimens such as BIC/FTC/TAF, vs TAF 10 mg, which is used with a booster as part of regimens such as DRV/c/FTC/TAF or EVG/c/FTC/TAF) and body mass index (BMI) or weight changes in a population of PLWH. In addition, the mechanism of action underlying ART-related weight gain or BMI increase remains unknown. Also unknown is whether a pharmacokinetic booster such as cobicistat might affect whether or not there is a relationship between TAF 10 mg and weight gain or BMI increase when compared with a higher dose of TAF (25 mg) without a booster.

The present study used electronic medical records (EMR) to evaluate weight-related outcomes among PLWH in the United States who were initiated on a PI-, INSTI-, or NNRTI-based ART regimen containing TAF 25 mg, TAF 10 mg, TDF, or not containing TAF or TDF agents.

## METHODS

### Data Source

EMR data from Decision Resources Group’s (DRG) Real World Data Repository (part of Clarivate) from July 17, 2017, to March 1, 2020, were used in the current study. DRG’s EMR data, which covers more than 65 million lives (including 107 274 PLWH), is primarily ambulatory and includes specialist and primary care visits. Patient information, encounters, written prescriptions, diagnoses, and vitals (including weight and BMI) are available. DRG’s Real World Data Repository includes patients from all states and is broadly representative of the entire US population. The data is deidentified and complies with the patient requirements of the Health Insurance Portability and Accountability Act.

### Study Design

A retrospective longitudinal study design was used whereby the date of initiation of a DHHS-recommended ART regimen containing TAF 25 mg (TAF 25 mg cohort), TAF 10 mg (TAF 10 mg cohort), TDF (TDF cohort), or non-TAF/TDF NRTIs (non-TAF/ TDF cohort) between July 17, 2018 (most recent date of approval for a TAF-based ART in the United States), and October 15, 2019, was defined as the index date. The list of DHHS-recommended ART regimens is included in **Supplementary Table 1**. For STRs, the index date was defined as the date of the prescription for the regimen. Patients treated with multiple-tablet regimens (MTRs) were included if the NRTI agent(s) were received with all required PI, INSTI, or NNRTI components within 14 days before or after the date of the prescription for the NRTI agent(s). For MTRs identified as part of the TAF 25 mg, TAF 10 mg, or TDF cohorts, the index date was defined as the date of the prescription for the TAF 25 mg, TAF 10 mg, or TDF agent used as part of the MTR. For MTRs identified as part of the non-TAF/TDF cohort, the index date was defined as the date of the prescription for the NRTI agent that completed the regimen.

Continuous clinical activity was defined as the period from the first to the last record in the EMR database. The start of continuous clinical activity was the date of the first record observed in any dataset part of the EMR for a given patient, while the end of continuous clinical activity was the date of the last observed record in any dataset part of the EMR for that patient. The 12-month period of continuous clinical activity preceding the index date was defined as the baseline period and the follow-up period spanned from the index date until the initiation of a new ART regimen that would result in the patient changing treatment cohort, end of continuous clinical activity or end of data availability, whichever occurred first.

### Study Population

**Figure 1.**
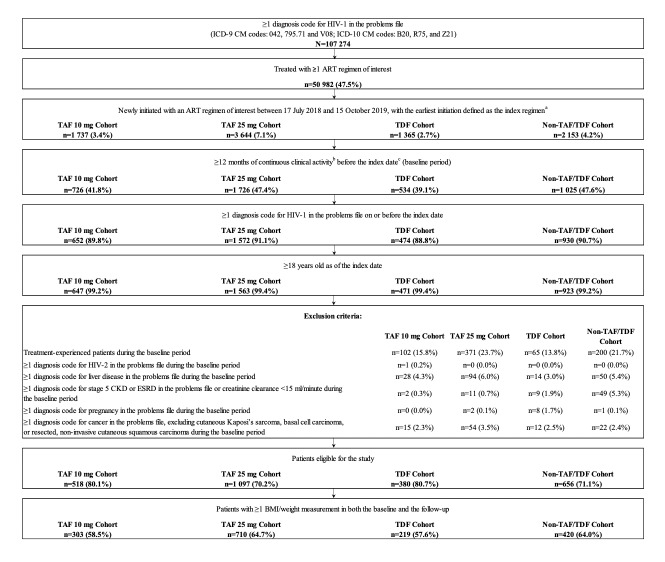
Identification of the Study Population Abbreviations: ART, antiretroviral therapy; BMI, body mass index; CKD, chronic kidney disease; EMR, electronic medical records; ESRD, end-stage renal disease; HIV, human immunodeficiency virus; ICD-9 CM/ICD-10 CM, *International Classification of Disease, Ninth/Tenth Revision, Clinical Modification*; TAF, tenofovir alafenamide; TDF, tenofovir disoproxil fumarate. ^a^ The index period spanned from July 17, 2018 (date of darunavir/cobicistat/emtricitabine/TAF approval), to October 15, 2019, to allow sufficient follow-up time to observe weight or BMI measurements during the observation period. ^b^ Continuous clinical activity was defined as the period from the first to last record in the EMR database. ^c^ For multi-tablet regimens (MTRs) identified as part of the TAF 10 mg, TAF 25 mg, or TDF cohorts, the index date was the date of the prescription for the TAF 10 mg, TAF 25 mg, or TDF agent used as part of the MTR. For MTRs identified as part of the non-TAF/TDF cohort, the index date was the date that regimen identification was complete.

Adult patients who initiated an ART regimen containing TAF 25 mg, TAF 10 mg, TDF, or non-TAF/TDF NRTIs between July 17, 2018, and October 15, 2019, were included if they had ≥1 diagnosis of HIV-1 on or before the index date, ≥12 months of continuous clinical activity before the index date, and ≥1 weight or BMI measurement in both the baseline and follow-up periods (Figure 1).

Patients were excluded if they were previously treated with an ART (ie, treatment-experienced) during the baseline period, or had ≥1 diagnosis of HIV-2, liver disease (including cirrhosis and hepatitis), chronic renal insufficiency (or creatinine clearance <15 mL/min), cancer (excluding cutaneous Kaposi’s sarcoma, basal cell carcinoma, or resected, noninvasive cutaneous squamous carcinoma), or pregnancy during the baseline period.

### Study Measures

Demographic and clinical characteristics were described during the 12-month baseline period. The weight or BMI measurement closest to the index date in the baseline period (or within 30 days post-index if no pre-index measurements were available) was defined as the pre-index weight or BMI measurement. The post-index weight or BMI measurement closest to the 3-, 6-, 9-, or 12-month time point (and within 45 days before or after the time point) was defined as the corresponding post-index measurement for that specific time point. For each post-index time point, the absolute and relative differences (ie, increase >0%, ≥5%, and ≥10%) in weight and BMI between the post-index time point and the pre-index measurement were assessed. To further understand the temporal trends in these changes, the time to weight or BMI increase of ≥5% or ≥10% was also evaluated over the entire follow-up period for all study cohorts.

### Statistical Analysis

Baseline characteristics were reported using means, standard deviations, and medians for continuous variables, and counts and proportions for categorical variables. Baseline characteristics were compared between the TAF 25 mg cohort and each other cohort using two-sample *t* test for continuous variables and chi-square test for categorical variables, with standardized differences for all variables.^12^

The mean change in weight and BMI between the pre- and post-index periods was compared between the TAF 25 mg cohort and each other cohort at 3, 6, 9, and 12 months post-index using mean differences, 95% CI, and *P* values obtained from adjusted ordinary least square regression models. The proportion of patients having any, ≥5%, and ≥10% weight and BMI increase between the post- and pre-index periods was compared between the TAF 25 mg cohort and all other cohorts at 3, 6, 9, and 12 months post-index using odds ratios, 95% CIs, and *P* values obtained from adjusted logistic regression models. Given that not all patients had a weight or BMI measurement at each study time point, the number of patients available for comparisons varied depending on the time point considered. Therefore, an analysis comparing the time to weight or BMI increase ≥5% and ≥10%, which included all patients, was performed between the TAF 25 mg cohort and each other cohort using hazard ratios, 95% CIs, and *P* values obtained from adjusted Cox proportional hazard models.

All regression models were adjusted for the following baseline characteristics: age, gender, race, insurance plan type, US geographic region, year of the index date, number of mental health-related comorbidities (see **Supplementary Table 2** for complete list of mental health-related comorbidities), symptomatic HIV/AIDS, Quan-Charlson Comorbidity Index (CCI) score,^13^ hypertension, type 2 diabetes, prediabetes, baseline BMI, medication class of the third agent used as part of the index ART regimen (ie, INSTI vs PI/NNRTI agents; the indicator was built as such since most patients used an INSTI as part of their index regimen), and use of a medication associated with weight change (see **Supplementary Table 3** for complete list of medications associated with weight change). The inclusion of variables that may predispose patients to gain weight (such as hypertension, type 2 diabetes, and prediabetes) were included as covariates as part of the multivariable adjustment to mitigate biases arising from confounding by indication, whereby patients at greater risk of weight gain may be more likely to be assigned medications known to have little impact on weight gain or BMI change. Within each regression model, the TAF 25 mg cohort was used as the reference group and all other NRTI cohorts were considered as comparison groups. The median time to weight or BMI increase ≥5% or ≥10% as well as the proportion of patients reaching each threshold at 12 months in each cohort was reported using a Kaplan-Meier analysis.

## RESULTS

A total of 1652 patients were eligible for inclusion in the study, including 710 in the TAF 25 mg cohort, 303 in the TAF 10 mg cohort, 219 in the TDF cohort, and 420 in the non-TAF/TDF cohort (Figure 1).

### Baseline Characteristics

Baseline characteristics for all four cohorts are presented in Table 1. The mean age ranged from 49.7 years in the TAF 25 mg cohort to 51.3 years in the non-TAF/TDF cohort. The proportion of female patients ranged from 26.5% in the TAF 25 mg cohort to 30.6% in the TDF cohort. In all cohorts, most patients were White (range, 33.3% in TAF 10 mg and TDF cohorts to 39.3% in TAF 25 mg cohort) or Black/African American (range, 28.6% in TAF 25 mg cohort to 35.0% in TAF 10 mg cohort), resided in the South (range, 55.8% in TAF 10 mg cohort to 58.7% in TAF 25 mg cohort), and were covered by commercial insurance plans (range, 64.3% in TDF cohort to 70.1% in non-TAF/TDF cohort). The mean baseline weight ranged from 83.6 kg (184.3 lb) in the non-TAF/TDF cohort to 85.1 kg (187.6 lb) in the TAF 10 mg cohort and the mean baseline BMI ranged from 28.0 kg/m^2^ in the non-TAF/TDF cohort to 28.9 kg/m^2^ in the TAF 10 mg cohort, with no statistically significant differences found between cohorts. Although baseline characteristics were generally similar between the cohorts, there were some differences between the TAF 25 mg cohort relative to the other cohorts. Most notably, as detected by a standardized difference >10% and *P* value <0.05, there were a higher proportion of patients initiating index treatment in 2018-2019 in the TAF 10 mg, TDF, and non-TAF/TDF cohorts than in the TAF 25 mg cohort. There were also a lower number of mental health–related comorbidities in the TAF 10 mg cohort than in the TAF 25 mg cohort.

**Table 1.** Baseline Characteristics During the 12-Month Period Prior to the Index Date

	**Cohort**				**Standardized Difference (*P* Value^a^) vs TAF 25 mg**					
	**TAF 25 mg (n=710)**	**TAF 10 mg (n=303)**	**TDF (n=219)**	**Non-TAF/TDF (n=420)**	**TAF 10 mg**		**TDF**		**Non-TAF/TDF**	
Age at index date (y), mean±SD [median]	49.7±13.7 [52.0]	50.2±12.8 [53.0]	49.9±13.2 [52.0]	51.3±13.0 [52.0]	3.6%	(0.61)	1.5%	(0.85)	11.6%	(0.06)
Age categories (y), n (%)										
18-24	28 (3.9)	8 (2.6)	3 (1.4)	5 (1.2)	7.3%	(0.61)	16.1%	(0.40)	17.5%	(0.10)
25-34	96 (13.5)	38 (12.5)	31 (14.2)	46 (11.0)	2.9%		1.8%		7.8%	
35-44	116 (16.3)	47 (15.5)	43 (19.6)	76 (18.1)	2.3%		8.6%		4.7%	
45-54	173 (24.4)	78 (25.7)	48 (21.9)	110 (26.2)	3.2%		5.8%		4.2%	
55-64	198 (27.9)	97 (32.0)	65 (29.7)	122 (29.0)	9.0%		4.0%		2.6%	
≥65	99 (13.9)	35 (11.6)	29 (13.2)	61 (14.5)	7.2%		2.0%		1.7%	
Female, n (%)	188 (26.5)	88 (29.0)	67 (30.6)	116 (27.6)	5.7%	(0.40)	9.1%	(0.23)	2.6%	(0.68)
Race, n (%)										
White	279 (39.3)	101 (33.3)	73 (33.3)	148 (35.2)	12.4%	(0.14)	12.4%	(0.59)	8.4%	(0.43)
Black	203 (28.6)	106 (35.0)	66 (30.1)	125 (29.8)	13.8%		3.4%		2.6%	
Hispanic	48 (6.8)	20 (6.6)	17 (7.8)	28 (6.7)	0.6%		3.9%		0.4%	
Other	20 (2.8)	4 (1.3)	6 (2.7)	8 (1.9)	10.5%		0.5%		6.0%	
Unknown	160 (22.5)	72 (23.8)	57 (26.0)	111 (26.4)	2.9%		8.2%		9.1%	
Insurance plan type, n (%)										
Insurance plan available	570 (80.3)	246 (81.2)	171 (78.1)	344 (81.9)	2.3%	(0.74)	5.4%	(0.48)	4.1%	(0.50)
Commercial	395 (69.3)	170 (69.1)	110 (64.3)	241 (70.1)	0.4%	(0.72)	10.6%	(0.12)	1.7%	(0.28)
Medicare	99 (17.4)	39 (15.9)	26 (15.2)	56 (16.3)	4.1%		5.9%		2.9%	
Medicaid	59 (10.4)	26 (10.6)	25 (14.6)	29 (8.4)	0.7%		12.9%		6.6%	
Other	17 (3.0)	11 (4.5)	10 (5.8)	18 (5.2)	7.9%		14.0%		11.4%	
US geographic region, n (%)										
South	417 (58.7)	169 (55.8)	126 (57.5)	240 (57.1)	6.0%	(0.81)	2.4%	(0.95)	3.2%	(0.85)
West	141 (19.9)	62 (20.5)	42 (19.2)	92 (21.9)	1.5%		1.7%		5.0%	
Northeast	82 (11.5)	39 (12.9)	28 (12.8)	48 (11.4)	4.0%		3.8%		0.4%	
Midwest	57 (8.0)	29 (9.6)	20 (9.1)	35 (8.3)	5.4%		3.9%		1.1%	
Unknown	13 (1.8)	4 (1.3)	3 (1.4)	5 (1.2)	4.1%		3.7%		5.3%	
Year of index date, n (%)										
2018	215 (30.3)	144 (47.5)	95 (43.4)	162 (38.6)	35.9%	(<0.001*)	27.4%	(<0.001*)	17.5%	(0.004*)
2019	495 (69.7)	159 (52.5)	124 (56.6)	258 (61.4)	35.9%		27.4%		17.5%	
Number of mental health-related comorbidities,^b^ mean±SD [median]	0.5±1.0 [0.0]	0.3±0.8 [0.0]	0.4±0.9 [0.0]	0.5±1.0 [0.0]	15.8%	(0.02*)	6.3%	(0.39)	1.8%	(0.76)
Symptomatic HIV and AIDS	193 (27.2)	77 (25.4)	52 (23.7)	124 (29.5)	4.0%	(0.56)	7.9%	(0.31)	5.2%	(0.40)
Quan-CCI,^c^ mean±SD [median]	1.9±2.9 [0.0]	1.8±2.9 [0.0]	1.7±2.9 [0.0]	2.2±3.2 [0.0]	2.7%	(0.69)	5.1%	(0.51)	10.5%	(0.09)
Hypertension	108 (15.2)	41 (13.5)	28 (12.8)	73 (17.4)	4.8%	(0.49)	7.0%	(0.38)	5.9%	(0.34)
Type 2 diabetes mellitus	38 (5.4)	25 (8.3)	12 (5.5)	25 (6.0)	11.5%	(0.08)	0.6%	(0.94)	2.6%	(0.67)
Pre-diabetes	17 (2.4)	5 (1.7)	6 (2.7)	9 (2.1)	5.3%	(0.46)	2.2%	(0.77)	1.7%	(0.79)
Patients with a BMI measurement, n (%)	703 (99.0)	299 (98.7)	217 (99.1)	411 (97.9)	3.1%	(0.64)	0.7%	(0.92)	9.3%	(0.11)
BMI (kg/m^2^), mean±SD [median]	28.3±6.2 [27.1]	28.9±8.9 [27.5]	28.5±6.1 [27.7]	28.0±5.8 [27.1]	8.2%	(0.27)	2.9%	(0.71)	4.4%	(0.48)
BMI categories (kg/m^2^), n (%)										
<25	225 (32.0)	92 (30.8)	64 (29.5)	131 (31.9)	2.7%	(0.92)	5.4%	(0.79)	0.3%	(0.56)
25-29	247 (35.1)	104 (34.8)	82 (37.8)	157 (38.2)	0.7%		5.5%		6.4%	
30-34	130 (18.5)	55 (18.4)	37 (17.1)	75 (18.2)	0.3%		3.8%		0.6%	
≥35	101 (14.4)	48 (16.1)	34 (15.7)	48 (11.7)	4.7%		3.6%		8.0%	
Patients with a weight measurement, n (%)	710 (100.0)	303 (100.0)	219 (100.0)	420 (100.0)	—	—	—	—	—	—
Weight (kg), mean±SD [median]	84.2±18.7 [82.1]	85.1±20.7 [81.9]	83.8±19.1 [81.9]	83.6±18.1 [81.7]	4.7%	(0.50)	1.9%	(0.81)	3.1%	(0.62)
Index regimen, n (%)										
PI-based	27 (3.8)	39 (12.9)	9 (4.1)	7 (1.7)	33.3%	(<0.001*)	1.6%	(0.84)	13.1%	(0.04*)
Darunavir-based	21 (3.0)	39 (12.9)	8 (3.7)	7 (1.7)	37.4%	(<0.001*)	3.9%	(0.61)	8.6%	(0.18)
Atazanavir-based	6 (0.8)	0 (0.0)	1 (0.5)	0 (0.0)	—	—	4.8%	(0.56)	—	—
INSTI-based	591 (83.2)	264 (87.1)	99 (45.2)	418 (99.5)	11.0%	(0.12)	86.4%	(<0.001*)	60.6%	(<0.001*)
Dolutegravir-based	102 (14.4)	0 (0.0)	19 (8.7)	413 (98.3)	—	—	17.9%	(0.03*)	318.0%	(<0.001*)
Elvitegravir-based	0 (0.0)	264 (87.1)	52 (23.7)	0 (0.0)	—	—	—	—	—	—
Raltegravir-based	19 (2.7)	0 (0.0)	28 (12.8)	5 (1.2)	—	—	38.5%	(<0.001*)	10.8%	(0.09)
Bictegravir-based	470 (66.2)	0 (0.0)	0 (0.0)	0 (0.0)	—	—	—	—	—	—
NNRTI-based	92 (13.0)	0 (0.0)	111 (50.7)	82 (19.5)	—	—	88.6%	(<0.001*)	17.9%	(0.003*)
Doravirine-based	0 (0.0)	0 (0.0)	0 (0.0)	0 (0.0)	—	—	—	—	—	—
Efavirenz-based	3 (0.4)	0 (0.0)	76 (34.7)	0 (0.0)	—	—	100.9%	(<0.001*)	—	—
Rilpivirine-based	89 (12.5)	0 (0.0)	35 (16.0)	82 (19.5)	—	—	9.9%	(0.19)	19.1%	(0.002*)
Use of at least one medication associated with weight change,^d^ n (%)	229 (32.3)	97 (32.0)	59 (26.9)	128 (30.5)	0.5%	(0.94)	11.7%	(0.14)	3.8%	(0.54)

* *P* value significant at the 5% level.

Abbreviations: BMI, body mass index; CCI, Charlson Comorbidity Index; INSTI, integrase strand transfer inhibitor; NNRTI, non-nucleoside reverse transcriptase inhibitor; PI, protease inhibitor; TAF, tenofovir alafenamide; TDF, tenofovir disoproxil fumarate.

^a^*P* values were obtained from chi-square test for categorical variables or *t* test for continuous variables. All chi-square tests used 1 degree of freedom (*df*), with the following exceptions: categorical age (*df*=5), race (*df*=4), insurance plan type (*df*=3), US geographic region (*df*=4), and BMI categories (*df*=3).

^b^ Based on the mental health–related comorbidities listed in the *Diagnostic and Statistical Manual of Mental Disorders, 5th Edition*. See **Supplementary Table 2** for the complete list of mental health-related comorbidities.

^c^ Based on the methodology described in Quan et al.^13^

^d^ See **Supplementary Table 3** for the full list of medications associated with weight change.

INSTI use as part of the index ART regimen was common in all cohorts, particularly in the TAF 25 mg (83.2%), TAF 10 mg (87.1%), and non-TAF/TDF cohorts (99.5%), where a majority of patients initiated an INSTI. The only exception was the TDF cohort, where an INSTI (45.2%) was the second most used ART class of medications after NNRTIs (50.7%). More specifically, in the TAF 25 mg cohort, 66.2% of patients initiated BIC/FTC/TAF and 14.4% initiated a DTG-based regimen. In the TAF 10 mg cohort, 87.1% initiated EVG/c/FTC/TAF and 12.9% initiated DRV/c/FTC/TAF. In the TDF cohort, the most common NNRTI-based regimens were efavirenz-based (34.7%) and rilpivirine-based (16.0%), while the most common INSTI-based regimens were EVG/c/FTC/TDF (23.7%), raltegravir-based (12.8%), and DTG-based (8.7%). In the non-TAF/TDF cohort, 98.3% initiated a DTG-based regimen (mostly DTG/abacavir/lamivudine [77.6%] and DTG/rilpivirine [15.7%]).

### Comparison of Weight and BMI Change at Specific Time Points

**Figure 2.**
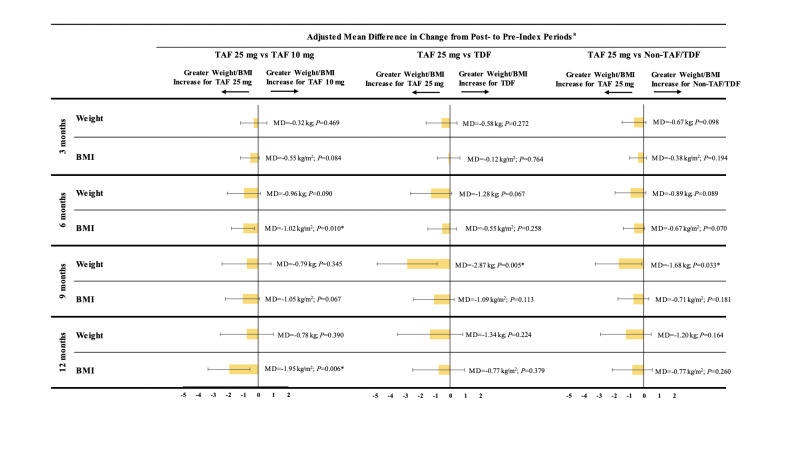
Comparison of Mean Weight or BMI Change Between Pre-index and Post-index Periods * *P* value significant at 5% level. Abbreviations: BMI, body mass index; CI, confidence interval; MD, mean difference; TAF, tenofovir alafenamide; TDF, tenofovir disoproxil fumarate. ^a^ Mean differences and their associated 95% CIs and P values were estimated using ordinary least squares regression models adjusted for the following baseline characteristics: age, gender, race, insurance plan type, US geographic region, year of the index date, number of mental health–related comorbidities, symptomatic HIV/AIDS, Quan-Charlson Comorbidity Index score, hypertension, type 2 diabetes, prediabetes, baseline BMI, use of an INSTI agent in the index regimen, and use of a medication associated with weight change. A mean difference <0 indicates that the TAF 10 mg, TDF, or non-TAF/TDF cohorts had a lower mean weight gain or BMI increase than the TAF 25 mg cohort.

Patients in the TAF 10 mg, TDF, and non-TAF/TDF cohorts experienced numerically smaller absolute weight increases than patients in the TAF 25 mg cohort across all time points, although the differences did not reach statistical significance at every post-index time point. More specifically, relative to the TAF 25 mg cohort, weight increases were numerically smaller in the TAF 10 mg cohort, with adjusted mean differences ranging from −0.32 kg [−0.71 lb] (TAF 10 mg Δ~3 months~= 0.42 kg [0.93 lb]; TAF 25 mg Δ~3 months~= 0.82 kg [1.81 lb]; *P*=0.469) at 3 months to −0.78 kg [−1.72 lb] (TAF 10 mg Δ~12 months~= 1.08 kg [2.38 lb]; TAF 25 mg Δ~12 months~= 1.72 kg [3.79 lb]; *P*=0.390) at 12 months. Similar results were observed for the comparison of the TAF 25 mg cohort with the TDF and non-TAF/TDF cohorts (see Figure 2 for comparative results and **Supplementary Table S4** for unadjusted descriptive results).

Similarly, when compared to patients in the TAF 25 mg cohort, patients in the TAF 10 mg cohort experienced numerically smaller BMI increases, with adjusted mean differences ranging from −0.55 kg/m^2^ (TAF 10 mg Δ~3 months~= −0.29 kg/m^2^; TAF 25 mg Δ~3 months~= 0.32 kg/m^2^; *P*=0.084) at 3 months to −1.95 kg/m^2^ (TAF 10 mg Δ~12 months~= −0.92 kg/m^2^; TAF 25 mg Δ~12 months~= 0.71 kg/m^2^; *P*=0.006) at 12 months. Similar results for changes in BMI were observed for the comparison of the TAF 25 mg cohort with the TDF and non-TAF/TDF cohorts (see Figure 2 for comparative results and **Supplementary Table S4** for unadjusted descriptive results).

**Figure 3.**
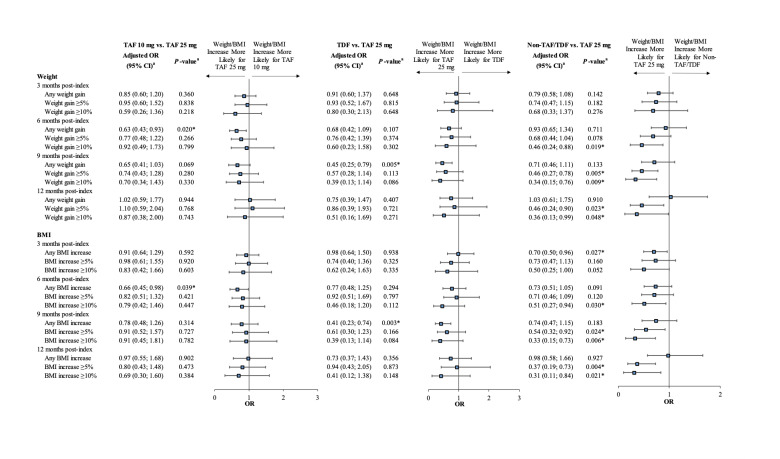
Comparison of Patients Having Any, ≥5%, Or ≥10% Weight or BMI Increases Between Pre-index and Post-index Periods * *P* value significant at the 5% level. Abbreviations: BMI, body mass index; CI, confidence interval; OR, odds ratio; TAF, tenofovir alafenamide; TDF, tenofovir disoproxil fumarate. ^a^ ORs and their associated 95% CIs and *P* values were estimated using logistic regression models adjusted for the following baseline characteristics: age, gender, race, insurance plan type, US geographic region, year of the index date, number of mental health–related comorbidities, symptomatic HIV/AIDS, Quan-Charlson Comorbidity Index score, hypertension, type 2 diabetes, prediabetes, baseline BMI, use of an INSTI agent in the index regimen, and use of a medication associated with weight change. An OR <1 indicates that the TAF 10 mg, TDF, or non-TAF/TDF cohorts had a lower risk of a weight gain or BMI increase than the TAF 25 mg cohort.

In addition, compared with patients in the TAF 25 mg cohort, patients in the TAF 10 mg cohort also experienced numerically lower relative weight and BMI increases using relative outcome measures (ie, weight/BMI increase >0%, ≥5%, or ≥10%) at all time points (all adjusted ORs <1.00). Similar results were observed for the TDF and non-TAF/TDF cohorts compared with the TAF 25 mg cohort (see Figure 3 for comparative results and **Supplementary Table S5** for unadjusted descriptive results).

### Time to Weight or BMI Increase

**Figure 4.**
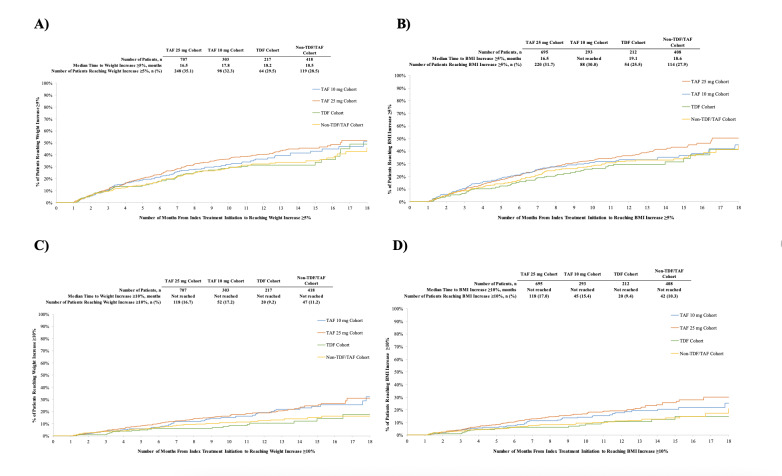
Kaplan-Meier Curves of Time to Weight Gain or BMI Increase ≥5% or ≥10% Abbreviations: BMI, body mass index; TAF, tenofovir alafenamide; TDF, tenofovir disoproxil fumarate.

The median time from index treatment initiation to weight gain ≥5% was shortest for the TAF 25 mg cohort (16.9 months) and longer for the TAF 10 mg, TDF, and non-TAF/TDF cohorts (Figure 4A). After 12 months, patients in the TAF 25 mg cohort had numerically higher Kaplan-Meier rates of weight increase ≥5% (40.1%) than patients in the TAF 10 mg, TDF, and non-TAF/TDF cohorts (31.3%-36.3%). Similar trends were observed for BMI increases, where the shortest median time to BMI increase ≥5% was found in the TAF 25 mg cohort (16.5 months; Figure 4B). After 12 months, patients in the TAF 25 mg cohort had numerically higher rates of BMI increase ≥5% (36.4%) than patients in the TAF 10 mg, TDF, and non-TAF/TDF cohorts (29.6%-33.1%). The median time to event was not reached in all cohorts for ≥10% weight increase (Figure 4C) or ≥10% BMI increase (Figure 4D).

**Figure 5.**
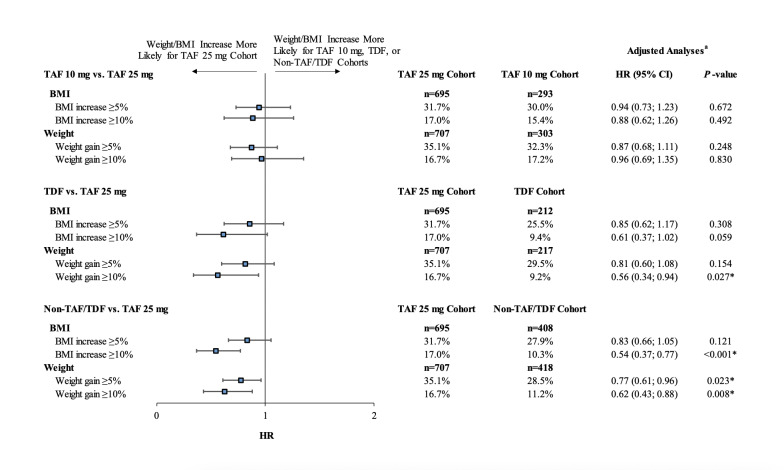
Comparison of Time to Weight Gain or BMI Increase ≥5% or ≥10% * *P* value significant at the 5% level. Abbreviations: BMI, body mass index; CI, confidence interval; HR, hazards ratio; TAF, tenofovir alafenamide; TDF, tenofovir disoproxil fumarate. ^a^ HRs and their associated 95% CIs and *P* values were estimated using Cox proportional hazards regression adjusted for the following baseline characteristics: age, gender, race, insurance plan type, US geographic region, year of the index date, number of mental health-related comorbidities, symptomatic HIV/AIDS, Quan-Charlson Comorbidity Index score, hypertension, type 2 diabetes, prediabetes, baseline BMI, use of an INSTI agent in the index regimen, and use of a medication associated with weight change. An HR <1 indicates that the TAF 10 mg, TDF, or non-TAF/TDF cohorts had a lower risk of weight gain or BMI increase than the TAF 25 mg cohort.

Over the entire follow-up period, patients initiated on a regimen containing TAF 10 mg, TDF, or non-TAF/TDF agents were less likely to experience weight increases of ≥5% or ≥10%, or BMI increases of ≥5% of ≥10%, compared with patients initiating TAF 25 mg (all adjusted hazard ratios <1.00), although the differences did not reach statistical significance for all comparisons (Figure 5).

## DISCUSSION

The current study, which predominantly included patients initiated on INSTIs, adds to the body of literature evaluating the impact of ART regimens containing different NRTIs at varying doses on changes in weight-related outcomes among PLWH in the United States. To our knowledge, this is the first real-world study to include differences in TAF doses as part of the analyses. While the fact that a majority of patients using TAF 25 mg in the current study were treated with BIC/ FTC/TAF and a majority of patients using TAF 10 mg were treated with EVG/c/FTC/TAF precludes any definitive conclusion about the impact different TAF doses may have on weight-related outcomes, this analysis does help isolate more of the impact that different TAF doses have on weight and BMI changes given the large proportion of INSTI users in both TAF treatment cohorts. In particular, results showed that the use of TAF 10 mg consistently had a smaller impact on weight and BMI increase than the use of TAF 25 mg, and findings reached statistical significance for some of the time points. Results also showed a similar trend in increased weight and BMI gain following initiation of ART regimens containing TAF 25 mg compared to TDF and non-TAF/TDF agents, with findings reaching statistical significance for a few additional outcomes measures and time points.

The present study, with its focus on the NRTI backbone, builds on other contemporary real-world studies in the US which have shown differential weight gain related to the third agent of ART regimens (eg, INSTI vs PI) among PLWH in routine clinical practice. Recently, a retrospective longitudinal study by Chow et al^14^ using administrative claims linked to EMR data found that over a mean follow-up period of 7 months, the PI cohort was 39% and 49% less likely to experience

≥5% weight and BMI increase than the INSTI cohort, respectively. Similarly, another recent study of treatment-naïve or virologically suppressed stable switchers by Emond et al^15^ using administrative claims linked to EMR found that patients initiated on BIC/FTC/TAF (an INSTI-based STR) had greater weight and BMI increases over a 1-year follow-up period than patients initiated on DRV/c/FTC/TAF (a PI-based STR). These prior studies not only help to corroborate the association between INSTI-based regimens and greater weight gain observed in multiple clinical trials^3,6^ but also among representative samples in real-world clinical practice. Of note, these prior EMR studies only accounted for the impact of the third agent, and not the NRTI backbone used, on weight changes among these populations.^14,15^ Thus, the present EMR study adds to the literature by showing that the use of different NRTIs and their doses may also be associated with variations in weight gain among real-world populations, although findings should also be interpreted in light of the specific third agent used.

In the current study, INSTIs were commonly used in all cohorts, although the specific INSTI agent used varied considerably. In addition, within the same cohort, further exploration of the study results showed that depending on the third agent used, weight and BMI increases were different. For instance, within the TAF 10 mg cohort, patients initiated on DRV/c/FTC/TAF (a PI-based STR) were found to have lower weight and BMI changes (-0.58 kg [−1.28 lb] and −1.24 kg/m^2^ at 12 months post-index) than those initiated on EVG/c/FTC/TAF (an INSTI-based STR; 1.23 kg [2.71 lb] and -0.89 kg/m^2^ at 12 months post-index), although the sample size was too small to make any statistical inference. Similar findings related to higher weight gain associated with INSTI agents, after controlling for the NRTI backbone, were observed in a prospective multicenter cohort study in Spain, which reported a mean increase of 3.5 kg [7.7 lb] for patients initiating an EVG-based regimen and 3.2 kg [7.0 lb] for patients initiating a DTG-based regimen at 36 months. The weight increase observed for patients treated with an EVG-based regimen was numerically higher than increases observed for patients treated with a PI- (mean increase of 3.2 kg [7.0 lb]) or NNRTI-based regimen (mean increase of 2.0 kg [4.4 lb]) over the same time period.^16^ In the same study, after adjusting for the third agent used, these observed increases were larger than observed weight increases related to using a backbone containing FTC/TAF (0.90 kg/year [2.0 lb/year]) relative to backbones containing FTC/TDF or abacavir/lamivudine.^16^ Yet while these data suggest a lower weight impact depending on the choice of ART backbone, evidence from the literature suggests that the combination of an INSTI with TAF may be associated with the highest weight gain.

As mentioned above and per the DHHS guidelines,^2^ data now suggest greater weight gain associated with certain INSTI-based regimens and TAF relative to other ART medications. This is also supported by our findings, which showed statistically significant higher weight gain and BMI increase for additional outcome measures and time points when comparing TAF 25 mg to TDF and non-TAF/TDF agents. More specifically focusing on the effect of TAF, a recent pooled analysis of randomized controlled clinical trials found that the initiation of FTC/TAF was associated with more weight gain than FTC/TDF and abacavir/lamivudine in treatment-naïve PLWH,^3^ which is consistent with the present study findings. Similarly, the ADVANCE trial of treatment-naïve patients with HIV-1 showed that a greater weight gain was associated with a DTG-based regimen when combined with TAF vs TDF.^6^ In a large recent study of 6908 ART-experienced, virologically suppressed PLWH in the US OPERA cohort, Mallon et al^17^ found that over a median follow-up period of approximately 20 months, switching from TDF to TAF was associated with early, pronounced weight gain (1.80 to 4.47 kg/year [3.97 to 9.85 lb/year]) among PLWH maintaining other ART regimens as well as those switching to an INSTI, irrespective of the INSTI agent used. Consistent with this, several small cohort studies outside the US have shown that a switch from TDF to TAF was associated with significant weight gain among patients treated with PI-, INSTI-, or NNRTI-based regimens during follow-up.^5,7,8,18^ Of note, while these prior cohort studies assessed the impact of switching from TDF to TAF on weight gain, they did not directly compare different TAF doses. While the above evidence shows that TAF may have an independent effect on weight gain compared with TDF, the magnitude of the effect may depend on type of patients studied (treatment-naïve or treatment-experienced) and may not be as important as the effect associated with the third agent. For instance, a recent EMR-based study of virologically suppressed INSTI-naïve patients in the United States who switched to either an INSTI- or non-INSTI-based ART regimen, demonstrated a rapid increase in BMI that was strongly associated with INSTI use in the first 8 months following switch, independent of concomitant TAF use, with only a slow increase in BMI after 8 months attributed solely to TAF.^19^ The clinical significance of all these findings warrants further investigation.^2^

The present findings may help inform future discussions surrounding the treatment and management of PLWH. In particular, the rising prevalence of weight gain and obesity among ART initiators^20^ is likely to add to the existing clinical burden among PLWH, potentially increasing the risk of chronic conditions such as diabetes and cardiovascular disease.^21–26^ This speaks to the need for considering ART-related weight gain when aiming to improve long-term prognosis among PLWH, especially for those with pre-existing risk factors. Of note, although weight continues to increase beyond the first year of treatment, approximately 80% of the weight gain observed at 3 years following the initiation of ART could be ascribed to the changes within the first year.^20^ As a result, weight gain should be among the factors considered for selection of an appropriate initial ART regimen for PLWH.

The present study was subject to certain limitations. First, although the study demonstrated some clear trends related to NRTI-associated weight gain, the specific time point analysis was likely underpowered to detect statistical significance for observed differences in weight and BMI changes between the TAF 25 mg cohort and each comparator cohort. Although the use of a time-to-event analysis that included all patients increased the study power, only larger reductions in the risk of weight or BMI increases of ≥5% and ≥10% were found to be statistically significant. Second, as mentioned above, since specific INSTI-based regimens were tied to the TAF 25 mg, TAF 10 mg, and non-TAF/TDF cohorts, it is difficult to separate the effect of specific NRTIs from the effect of specific INSTI agents on weight-related outcomes. Third, as with many studies using EMR, the data may contain inaccuracies or omissions. For instance, written prescriptions for ART regimens may not reflect actual use, since patients may not take medications as prescribed. In addition, viral load and CD4 cell count measurements, which have also been associated with weight change,^3^ were not available for the majority of patients and therefore could not be adjusted for in the analyses. Furthermore, creatinine clearance was not available for all patients; however, the absence of any evidence for this exclusion criterion (creatinine clearance <15 mL/min) does not guarantee that a patient did not have chronic renal insufficiency. Fourth, unlike claims-based data, where continuous periods of insurance eligibility can be identified, periods of continuous clinical activity in the EMR were approximated by using the dates of the first and last records in the database, and thus patients with large gaps in care may be incorrectly identified as having continuous activity. Finally, a limitation specific to provider-based data sources such as DRG is that they may not capture the services patients received from a provider that is outside the network.

## CONCLUSIONS

This retrospective longitudinal study of patients predominantly initiated on INSTIs compared weight-related outcomes among PLWH initiated on ART regimens containing TAF 25 mg (including BIC/FTC/TAF), TAF 10 mg (including DRV/c/FTC/ TAF and EVG/c/FTC/TAF), TDF, and non-TAF/TDF NRTIs. A trend toward less pronounced weight gain and BMI increases between the pre- and post-index period was observed among patients initiated on TAF 10 mg, TDF, and non-TAF/TDF NRTIs relative to those initiated on a TAF 25 mg, although these findings did not reach statistical significance for each comparison at all time points. Future studies with larger sample sizes and the ability to adjust for the use of specific PI, INSTI, or NNRTI agents will be needed to better understand the variations in weight-related outcomes associated with different NRTIs and TAF doses.

## Supplementary Material

Online Supplementary Material

Online Supplementary Material
