# Supplementary material for: Real-World Analysis of Weight Gain and Body Mass Index Increase Among Patients with HIV-1 Using Antiretroviral Regimen Containing Tenofovir Alafenamide, Tenofovir Disoproxil Fumarate, or Neither in the United States
Source: J Health Econ Outcomes Res. 2022 Feb 14;9(1):39–49. doi: 10.36469/jheor.2022.31825 (PMC8843358; doi:10.36469/jheor.2022.31825)
Supplement: Online Supplementary Material [file jheor_2022_9_1_31825_82308.pdf]

Journal of Health Economics and Outcomes Research

Online Supplementary Material

Real-World Analysis of Weight Gain and Body Mass Index Increase Among Patients With HIV-1 Using Antiretroviral Regimen Containing Tenofovir Alafenamide, Tenofovir Disoproxil Fumarate, or Neither in the United States. *JHEOR*. 2022;9(1):39-49. doi:10.36469/jheor.2022.31825

Table S1: ART Regimens Included in Each Study Cohort

Table S2: Mental Health–Related Comorbidities

Table S3: Medication Classes and Agents Associated With Weight Change

Table S4: Unadjusted Mean Weight and BMI in Pre- and Post-index Periods

Table S5: Unadjusted Proportions of Patients Having Any, ≥5%, or ≥10% Weight or BMI Increases Between Pre- and Post-Index Periods

This supplementary material has been provided by the authors to give readers additional information about their work.

This is an open-access article distributed under the terms of the Creative Commons Attribution 4.0 International License (CCBY-4.0). View this license's legal deed at http://creativecommons.org/licenses/by/4.0 and legal code at http://creativecommons.org/licenses/by/4.0/legalcode for more information.

Table S1. ART Regimens Included in Each Study Cohort

TAF 10 mg	TAF 25 mg	TDF	Non-TDF/TAF
Darunavir/cobicistat/FTC/TAF (STR)	Bictegravir/FTC/TAF (STR)	Elvitegravir/cobicistat/FTC/TDF (STR)	Dolutegravir/lamivudine (STR)
Elvitegravir/cobicistat/FTC/TAF (STR)	Dolutegravir + FTC/TAF (25 mg) (MTR)	Dolutegravir + FTC/TDF (MTR)	Dolutegravir/abacavir/lamivudine (STR)
	Raltegravir + FTC/TAF (25 mg) (MTR)	Dolutegravir + TDF + lamivudine (MTR)	Dolutegravir/rilpivirine (STR) or dolutegravir + rilpivirine (MTR)
	Darunavir/cobicistat + FTC/TAF (25 mg) (MTR)	Raltegravir + FTC/TDF (MTR)	Darunavir/cobicistat + abacavir/lamivudine (MTR)
	Atazanavir/cobicistat + FTC/TAF (25 mg) (MTR)	Raltegravir + TDF + lamivudine (MTR)	Darunavir/cobicistat + lamivudine (MTR)
	Doravirine + FTC/TAF (25 mg) (MTR)	Darunavir/cobicistat + FTC/TDF (MTR)	Darunavir/cobicistat + raltegravir (MTR)
	Rilpivirine/FTC/TAF (STR)	Darunavir/cobicistat + TDF + lamivudine (MTR)	
	Efavirenz + FTC/TAF (25 mg) (MTR)	Atazanavir/cobicistat + FTC/TDF (MTR)	
		Atazanavir/cobicistat + TDF + lamivudine (MTR)	
		Doravirine/TDF/lamivudine (STR) or Doravirine + TDF/lamivudine (MTR)	
		Rilpivirine/FTC/TDF (STR)	
		Efavirenz/FTC/TDF (STR) or Efavirenz + FTC/TDF (MTR)	
		Efavirenz/TDF/lamivudine (STR)	

Abbreviations: ART, antiretroviral therapy; FTC, emtricitabine; MTR, multiple-tablet regimen; STR, single-tablet regimen; TAF, tenofovir alafenamide; TDF, tenofovir disoproxil fumarate.

Table S2. Mental Health–Related Comorbidities

Anxiety disorders	Other conditions that may require a focus of clinical attention
Bipolar and related disorders	Other mental disorders
Depressive disorders	Paraphilic disorders
Disruptive, impulse-control, and conduct disorders	Personality disorders
Dissociative disorders	Schizophrenia spectrum and other psychotic disorders
Elimination disorders	Sexual dysfunctions
Feeding and eating disorders	Sleep-wake disorders
Gender dysphoria	Somatic symptom and related disorders
Medication-induced movement disorders and other adverse effects of medication	Substance-related and addictive disorders
Neurocognitive disorders	Trauma- and stressor-related disorders
Neurodevelopmental disorders	
Obsessive-compulsive and related disorders	

Source: American Psychiatric Association. *Diagnostic and Statistical Manual of Mental Disorders (Fifth Edition),* 2013.

Table S3. Medication Classes and Agents Associated With Weight Change

Antidiabetics
Biguanides/meglitinides
Insulins
Sulfonylureas
Dipeptidyl peptidase-4 (DPP-4) inhibitors
Thiazolidinediones
Glucagon-like peptide-1 (GLP-1) agonists
α-Glucosidase inhibitors
Other antidiabetic medications
Antihyperlipidemics
Antihypertensives
Appetite stimulants/suppressants
Amphetamine/dextroamphetamine
Phentermine
Dronabinol
Methylphenidate
Megestrol
Oxandrolone
Topiramate
β-Blockers
Corticosteroids
Hormonal therapies
Psychiatric/neurologic therapies
Antidepressants
Anticonvulsants
Antipsychotics

Table S4. Unadjusted Mean Weight and BMI in Pre- and Post-index Periods

	TAF 25 mg Cohort	TAF 10 mg Cohort	TDF Cohort	Non-TAF/TDF Cohort
**3 Months**				
Weight				
Patients with both baseline and follow-up measurements, n	n=477	n=201	n=140	n=271
Weight (kg) pre-index, mean±SD [median]	84.37±18.90 [81.92]	84.92±21.10 [81.48]	83.11±19.06 [81.79]	83.43±18.85 [81.92]
Weight (kg) at 3 months post-index, mean±SD [median]	85.20±18.90 [83.00]	85.34±21.34 [83.01]	83.04±17.74 [83.28]	83.71±18.88 [81.92]
BMI				
Patients with both baseline and follow-up measurements, n	n=467	n=196	n=136	n=266
BMI (kg/m^2) pre-index, mean±SD [median]	28.24±6.20 [27.12]	29.08±10.10 [27.22]	28.26±5.92 [27.60]	27.93±5.97 [26.95]
BMI (kg/m^2) at 3 months post-index, mean±SD [median]	28.57±6.30 [27.26]	28.79±6.49 [27.23]	28.28±5.65 [27.53]	27.94±5.72 [27.13]
**6 Months**				
Weight				
Patients with both baseline and follow-up measurements, n	n=391	n=164	n=108	n=216
Weight (kg) pre-index, mean±SD [median]	83.83±19.06 [83.07]	85.36±20.71 [83.00]	85.77±20.49 [84.24]	82.95±17.27 [81.57]
Weight (kg) at 6 months post-index, mean±SD [median]	85.55±19.01 [84.14]	86.05±21.40 [84.19]	85.63±19.38 [83.47]	83.84±17.63 [81.85]
BMI				
Patients with both baseline and follow-up measurements, n	n=381	n=159	n=106	n=210
BMI (kg/m^2) pre-index, mean±SD [median]	28.46±6.41 [27.12]	29.38±10.79 [27.90]	29.31±6.39 [28.15]	27.68±5.65 [26.97]
BMI (kg/m^2) at 6 months post-index, mean±SD [median]	29.13±6.52 [28.03]	29.06±6.63 [27.91]	29.21±6.25 [28.03]	27.81±5.39 [27.20]
**9 Months**				
Weight				
Patients with both baseline and follow-up measurements, n	n=254	n=116	n=82	n=153
Weight (kg) pre-index, mean±SD [median]	84.28±19.86 [82.62]	85.53±20.23 [81.92]	81.99±18.14 [80.92]	84.77±18.87 [81.92]
Weight (kg) at 9 months post-index, mean±SD [median]	86.47±19.91 [84.63]	86.63±20.72 [84.04]	81.15±17.28 [79.67]	85.03±19.52 [81.81]
BMI				
Patients with both baseline and follow-up measurements, n	n=253	n=109	n=78	n=151
BMI (kg/m^2) pre-index, mean±SD [median]	28.67±6.53 [27.13]	29.68±12.34 [26.99]	27.70±5.39 [26.97]	28.52±6.52 [27.26]
BMI (kg/m^2) at 9 months post-index, mean±SD [median]	29.45±6.57 [28.24]	29.34±6.76 [27.90]	27.46±5.19 [26.65]	28.61±6.30 [27.28]
**12 Months**				
Weight				
Patients with both baseline and follow-up measurements, n	n=179	n=86	n=55	n=108

Weight (kg) pre-index, mean±SD [median]	84.26±20.02 [80.96]	87.08±20.41 [83.84]	82.79±20.23 [81.10]	83.94±18.40 [81.57]
Weight (kg) at 12 months post-index, mean±SD [median]	85.99±20.16 [83.01]	88.16±20.82 [85.27]	82.59±20.27 [81.92]	84.79±18.71 [81.51]
BMI				
Patients with both baseline and follow-up measurements, n	n=178	n=85	n=55	n=105
BMI (kg/m^2) pre-index, mean±SD [median]	28.35±6.40 [26.81]	30.06±13.50 [28.12]	27.60±6.07 [26.80]	28.37±6.14 [27.06]
BMI (kg/m^2) at 12 months post-index, mean±SD [median]	29.06±6.63 [27.18]	29.14±6.57 [28.16]	27.52±5.87 [26.82]	28.59±6.08 [27.03]

Abbreviations: BMI, body mass index; TAF, tenofovir alafenamide; TDF, tenofovir disoproxil fumarate.

Table S5. Unadjusted Proportions of Patients Having Any, ≥5%, or ≥10% Weight or BMI Increases Between Pre- and Post-Index Periods

	TAF 25 mg Cohort	TAF 10 mg Cohort	TDF Cohort	Non-TAF/TDF Cohort
**Weight**				
3 months post-index	**n=477**	**n=201**	**n=140**	**n=271**
Any weight gain	52.0%	47.8%	47.9%	47.2%
Weight gain ≥5%	17.6%	16.4%	15.0%	14.0%
Weight gain ≥10%	6.9%	4.0%	4.3%	4.8%
6 months post-index	**n=391**	**n=164**	**n=108**	**n=216**
Any weight gain	57.0%	46.3%	45.4%	55.6%
Weight gain ≥5%	26.1%	21.3%	19.4%	19.9%
Weight gain ≥10%	12.0%	11.0%	5.6%	6.9%
9 months post-index	**n=254**	**n=116**	**n=82**	**n=153**
Any weight gain	61.4%	48.3%	43.9%	50.3%
Weight gain ≥5%	32.7%	25.0%	19.5%	17.6%
Weight gain ≥10%	16.1%	11.2%	6.1%	5.9%
12 months post-index	**n=179**	**n=86**	**n=55**	**n=108**
Any weight gain	55.3%	54.7%	47.3%	56.5%
Weight gain ≥5%	28.5%	29.1%	21.8%	15.7%
Weight gain ≥10%	15.1%	12.8%	7.3%	5.6%
**BMI**				
3 months post-index	**n=467**	**n=196**	**n=136**	**n=266**
Any BMI increase	51.4%	48.5%	47.8%	44.4%
BMI increase ≥5%	18.8%	17.9%	13.2%	15.0%
BMI increase ≥10%	8.4%	6.6%	4.4%	4.5%
6 months post-index	**n=381**	**n=159**	**n=106**	**n=210**
Any BMI increase	57.7%	48.4%	49.1%	51.0%
BMI increase ≥5%	25.7%	22.0%	19.8%	21.0%
BMI increase ≥10%	13.4%	11.3%	5.7%	8.1%
9 months post-index	**n=253**	**n=109**	**n=78**	**n=151**
Any BMI increase	61.3%	52.3%	41.0%	51.7%
BMI increase ≥5%	33.2%	27.5%	20.5%	20.5%
BMI increase ≥10%	17.4%	13.8%	6.4%	6.6%
12 months post-index	**n=178**	**n=85**	**n=55**	**n=105**
Any BMI increase	56.2%	54.1%	47.3%	55.2%
BMI increase ≥5%	30.9%	27.1%	25.5%	15.2%
BMI increase ≥10%	16.3%	11.8%	7.3%	5.7%

Abbreviations: BMI, body mass index; TAF, tenofovir alafenamide; TDF, tenofovir disoproxil fumarate.
